# Turkish Adaptation of the Nurses' Perceptions of Electronic Documentation Scale

**DOI:** 10.1002/nop2.70547

**Published:** 2026-04-22

**Authors:** Sevgi Pakiş Çetin, Nurcan Bilgin

**Affiliations:** ^1^ Department of Fundamentals Nursing Manisa Celal Bayar University Manisa Turkey; ^2^ Department of Nursing Management Manisa Celal Bayar University Manisa Turkey

**Keywords:** electronic health records, NPED scale, nursing, reliability and validity

## Abstract

**Aim:**

This study was conducted to test the reliability and validity of the Nurses' Perceptions of Electronic Documentation (NPED) scale on Turkish nurses.

**Design:**

A methodological study.

**Methods:**

In the analysis, the scale's reliability was evaluated using Cronbach's alpha coefficient, item‐total score correlations, scale response bias and the test–retest method, in addition to descriptive statistics. Validity analyses were conducted through language validity, the content validity index, construct validity (Confirmatory Factor Analysis [CFA] and Exploratory Factor Analysis [EFA]) and known‐groups validity.

**Results:**

For the 11‐item NPED, item‐total score correlations ranged between 0.27 and 0.68 (*p* < 0.01). EFA was conducted due to poor goodness‐of‐fit indices observed in the initial CFA. The EFA identified three subfactors; however, items 2 and 10 were distributed in subdimensions different from the original scale. After removing items 2 and 10, the scale demonstrated acceptable results in EFA. While α = 0.795 in the NPED, Factor 1 (α = 0.855), Factor 2 (α = 0.741) and Factor 3 (α = 0.767) measured 73.48% of the total variance. In the known‐groups validity analysis, the scale successfully identified differences based on nurses' self‐reported proficiency in computer use and their prior exposure to Electronic Health Records (EHRs) training, both of which were established as criteria.

## 1 Introduction

The integration of new technologies in healthcare services has a significant impact on healthcare professionals and the delivery of nursing care (Schenk et al. 2020). Technology in nursing has brought about substantial transformations, offering numerous benefits that enhance patient care, streamline processes and improve healthcare delivery. One of the most notable advantages of incorporating technology into nursing practice is the use of Electronic Health Records (EHRs), which contribute to enhanced patient care and safety (Malla and Amin 2023). EHRs are digital records created within the healthcare environment that systematically compile patients' personal and clinical health data, functioning as a digital version of traditional written documents (Bani Issa et al. 2020; Bergier et al. 2021).

EHRs are real‐time, patient‐centred systems that provide immediate and safe access to patient information for authorised users and healthcare providers (Bergier et al. 2021). These records allow nurses to maintain comprehensive and up‐to‐date patient data, significantly reducing the risk of errors associated with manual recording or miscommunication among care providers (Rossi et al. 2023; Malla and Amin 2023). Furthermore, EHRs can protect nurses' rights in the event of complaints while also serving as evidence of the quality of nursing care delivered (Shan et al. 2023).

EHRs encompass a broad range of information, including patient demographics, prescriptions, diagnoses, vital signs, immunisations, laboratory and radiology results, medical concepts and notes, procedures and treatment plans. Comprehensive data within EHRs contributes to the enhancement of healthcare service quality in several key ways (Sarwar et al. 2022), including:

- Facilitating seamless data sharing among multiple healthcare organisations, such as research laboratories, specialists, medical imaging centres, pharmacies, emergency departments and medical schools.
- Providing healthcare providers with access to real‐time data and tools to help them make decisions about patient care plans.
- Tracking data better over time.
- Automating workflow for clinicians.
- Providing timely reminders for patient screenings and preventive checks to improve patient care.
- Facilitating research by hosting patients' medical history and healthcare data (Sarwar et al. 2022).
- Creating a reliable database for statistical evaluations and research by ensuring accurate and consistent maintenance of patient records (Gürkan et al. 2023).

Nurses represent the largest and most indispensable element of the healthcare team, providing primary healthcare services to patients, determining the needs of patients, providing individualised nursing care and questioning the adequacy of tools and materials. Therefore, nurses constitute the most valuable users of EHRs (Gürkan et al. 2023). Additionally, documenting nursing care on the EHRs is one of the professional responsibilities of nurses (Laukvik et al. 2023). EHRs are important in terms of enabling nurses to continuously reflect on their intervention choices for patients and the effects of the interventions they implement. Therefore, they are vital for quality, reliability, continuity and evidence‐based nursing care (De Groot et al. 2022; Laukvik et al. 2023).

Studies investigating the effects of EHRs on patient care and healthcare quality are prominent in the literature. Upadhyay and Hu (2022) reported that nurses found EHRs beneficial due to their convenience, particularly in checking drug side effects or interactions before administration and preventing challenges during doctors' absences or night shifts (Upadhyay and Hu 2022). Similarly, Schenk et al. (2021) highlighted that EHRs facilitated documentation, enhanced ease of use and improved patient care (Schenk et al. 2021). In a study by Alhur (2023), 65.3% of nurses acknowledged that EHRs were advantageous for their profession (Alhur 2023). Rossi et al. (2023), in their evaluation of EHRs for nursing care plans, concluded that they enabled nurses to reflect clinical judgement and make nursing care more visible (Rossi et al. 2023). Furthermore, Engin and Karatana (2025) reported that the use of EHRs increases the reliability of patient care, improves the quality of care and enhances the support provided to patients (Engin and Karatana 2025).

Schenk et al. (2020) developed the Nurses' Perceptions of Electronic Documentation (NPED) scale to assess nurses' perceptions of EHRs. This study was initiated due to the lack of a valid and reliable measurement tool in Türkiye for nurses, who are key users of EHRs. The use of EHRs by nurses is expected to enhance the quality of healthcare services by facilitating workflow and improving time management.

## 2 The Study

### 2.1 Aim

This study aimed to test the reliability and validity of the NPED scale among Turkish nurses.

## 3 Methods

### 3.1 Research Type and Sample

This methodological study was conducted with 211 nurses employed at a university hospital in western Türkiye. The general guideline for determining sample size recommends a minimum of five observations per variable, with a preferable ratio of 10:1 (Hair et al. 2019). Given the 11 items in the scale and the relevant literature, the minimum required sample size for this study was 110 participants. In addition, while some studies recommend minimum absolute sample sizes (e.g., *N* = 200 or 300) regardless of the number of variables, others emphasise that sample adequacy in factor analysis should be evaluated based on data‐dependent characteristics such as the magnitude and distribution of factor loadings, communalities and the number of extracted factors (Gunawan et al. 2021; Farag et al. 2025). The literature further indicates that recommended sample sizes for factor analysis range from as few as 50 to more than 1.000 participants, with suggested item‐to‐response ratios varying between 1:3 and 1:20 (Gunawan et al. 2021). Accordingly, data were collected from 211 participants for an 11‐item scale, yielding an approximate item‐to‐response ratio of 1:19.

The sample consisted of 211 nurses aged between 22 and 56 years who worked at a university hospital and agreed to participate in the study. Data collection took place between September and December 2024. A convenience sampling method based on voluntary participation was used. Data were collected through face‐to‐face distribution of printed survey forms, which were retrieved within a few days. All participants provided informed consent prior to inclusion. The survey was conducted anonymously, and no identifying information was collected.

### 3.2 Data Collection Tools

#### 3.2.1 Descriptive Information Form

Created by researchers based on relevant literature (Schenk et al. 2020, 2021; Brunelli et al. 2021), the form consists of 10 questions addressing demographic and professional information, including age, gender, marital status, educational background and employment unit.

#### 3.2.2 NPED

The NPED, developed by Schenk et al. (2020), measures nurses' perceptions of EHRs. The scale comprises 11 items and is structured as a 5‐point Likert scale. Of these, five items (items 2, 6, 7, 8, 9) are reverse‐coded. The scale has three subdimensions: ease of use (items 1, 2, 3, 4, 5, 10, 11), impacts of EHRs on nursing (items 6 and 7), and concerns about EHRs (items 8 and 9). A higher total score on the scale indicates a more positive perception of EHRs (Schenk et al. 2020). Cronbach's Alpha reliability coefficients reported by Schenk et al. (2020) were 0.84 for the ease of use subdimension, 0.82 for the impacts of EHRs on nursing and 0.85 for the concerns about EHRs subdimension.

### 3.3 Procedure

#### 3.3.1 Language and Content Validity of NPED

To ensure linguistic validity, the NPED scale was translated from English to Turkish by five independent individuals fluent in both languages. The researchers reviewed and consolidated these translations to create the initial Turkish version of the scale. The Turkish version was then translated back into English by an independent translator fluent in both languages. The back‐translated version was compared with the original scale by an expert proficient in English. The researchers evaluated the compatibility between the original scale and the back‐translated version, discussing each item in detail. Based on these evaluations, the final Turkish version of the scale was refined and accepted.

After completing the language adaptation process, the Davis technique was utilised to assess the content validity of the scale items. The Davis technique evaluates expert opinions on a four‐point scale: 1 = Not relevant, 2 = Somewhat relevant, 3 = Quite relevant and 4 = Very relevant. The Content Validity Index for each item (I‐CVI) was calculated by dividing the number of experts who rated the item as 3 or 4 by the total number of experts. To determine the content validity of the overall scale (S‐CVI), the average of the item‐level CVIs (I‐CVIs) was calculated (Grant and Davis 1997; Polit and Beck 2021). The Turkish version of the scale was reviewed by seven faculty members with expertise in diverse areas (including Fundamentals of Nursing, Public Health, Psychiatry, Internal Medicine, Paediatrics, Child Health, Obstetrics and Gynaecology and Nursing). These faculty members evaluated each item conceptually and rated it between 1 and 4 according to the Davis technique. The S‐CVI equals 0.90, above the recommended threshold of 0.80, indicating strong content validity (Madadizadeh and Bahariniya 2023). In this study, CVI was determined to be 0.90 for 11 items, which was considered acceptable as it exceeded the recommended threshold of 0.80. A preapplication of the final version of the scale was conducted with 10 participants, who assessed the clarity and comprehensibility of the items. Since no negative feedback was received, the data collection phase proceeded, and the responses from the preapplication participants were included in the analysis.

### 3.4 Data Analysis

Statistical analysis was conducted using IBM SPSS (Version 29.0) and LISREL software (IBM Corp 2022; Jöreskog and Sörbom 2025). Descriptive statistics were computed to describe demographic information. Kurtosis and skewness values were assessed to examine the normality of the data distribution (NPED: Kurtosis = −0.310, Skewness = 0.041). Kurtosis and skewness values between −1.5 and +1.5 indicate a normal distribution of the data (Tabachnick and Fidell 2021). The distribution of responses on the scale was further analysed by calculating the mean, standard error and standard deviation for the 11 items based on the nurses' responses. The scale's reliability was assessed using Cronbach's alpha coefficient, item‐total score correlation and scale response bias. In addition, the test–retest method was conducted using data collected from a subgroup of 30 participants. Cronbach's alpha values greater than 0.70 were considered to indicate adequate reliability (Çokluk et al. 2025; Taber 2018). Additionally, Cronbach's alpha values were recalculated and evaluated for each item when removed. Item‐total correlation values, which are generally expected to be above 0.3, were also computed (Hajjar 2018). The test–retest correlation values were interpreted according to the following scale: very weak correlation (0.00–0.10), weak correlation (0.10–0.39), moderate correlation (0.40–0.69), strong correlation (0.70–0.89) and very strong correlation (0.90–1.00) (Schober et al. 2018). The validity of the scale was assessed through language validity, CVI, construct validity (via Confirmatory Factor Analysis [CFA] and Exploratory Factor Analysis [EFA]) and known‐groups validity. It is recommended in the literature to conduct CFA in cross‐cultural scale adaptation studies; however, if CFA does not confirm the model structure of the original scale or if the model‐data fit is inadequate, EFA should be performed (Çokluk et al. 2025). For the present study, the three‐dimensional model from the original scale was evaluated using CFA. The following indices were reported: Chi‐square/degree of freedom (*χ* ^2^/df), Root Mean Square Error of Approximation (RMSEA), Root mean square residual (RMR), Standardised Root Mean Residual (SRMR), Goodness‐of‐Fit Index (GFI), Adjusted‐goodness‐of‐Fit Index (AGFI), Comparative Fit Index (CFI), Normed Fit Index (NFI) and Nonnormed Fit Index (NNFI). Many fit and cut‐off points have been specified in the literature for these values. A *χ* ^2^/df value of less than 5, RMSEA, RMR and SRMR below 0.08 and a CFI and NFI above 0.90 and GFI above 0.95 are considered indicators that the model has an acceptable fit (Whittaker and Schumacker 2022). For the known groups' validity, the differences in proficiency in computer use, the status of being informed about the EHRs in the past and the differences in the total score of the NPED scale were compared.

### 3.5 Ethical Considerations

This study adheres to the ethical principles outlined in the Declaration of Helsinki. For the adaptation of the measurement tool into Turkish, permission was obtained from Schenk et al. (2020) via e‐mail. For the implementation of the study, ethics committee permission (decision date: 20 December 2023; decision no: 20.478.486/2142) was obtained from the Health Sciences Ethics Committee of the Faculty of Medicine at Manisa Celal Bayar University, and permission was obtained from the institution where the data were collected. In addition, written permission was obtained from the nurses who agreed to participate in the study with the ‘Informed Voluntary Consent Form’.

## 4 Results

### 4.1 Characteristics of the Sample

The mean age of the nurses was 34.38 ± 7.20 years (22.00–56.00). Among them, 85.3% were female, 71.6% were married and 56.4% held a bachelor's degree. Regarding work settings, 37% were employed in internal units, 79.6% worked as clinical nurses, 64% worked more than 40 h per week and 75.4% worked in shifts. Additionally, 62.1% of the nurses reported their computer proficiency as ‘proficient’, while 52.6% stated they had not received prior training on EHRs.

### 4.2 Descriptive Statistics and Reliability Analyses of the Scale

The 11‐item NPED scale had a mean total score of 39.16 ± 5.59 (Min‐Max: 24.00–54.00), with subscale mean scores of 24.29 ± 3.90 for ease of use, 7.33 ± 1.89 for impacts of EHRs on nursing, and 7.54 ± 1.68 for concerns about EHRs. Mean item scores ranged from 2.79 to 3.93, and item‐total score correlations were between 0.27 and 0.68 (*p* < 0.01). The overall Cronbach's alpha coefficient was 0.75, with subscale reliability coefficients of 0.71, 0.76 and 0.74, respectively. Removing items 2 and 10, part of the ease of use subscale, resulted in a minimal increase in the overall Cronbach's alpha value to 0.77. In the case of item 2 deletion in the ease of use subscale, the internal consistency coefficient of the subscale increased to 0.81, but no significant correlation was found with the subscale's total score (*p* = 0.136). In contrast, deleting item 10 increased the internal consistency coefficient to 0.73 and revealed a moderate positive correlation with the subscale total score (*r* = 0.474; *p* = 0.000).

The Hotelling's *T* ^2^ test was conducted to examine whether the mean scores of the scale items differed from each other. The test result was significant (*T* ^2^ = 25.884, *p* < 0.001).

Pearson correlation analysis, conducted at 4‐week intervals on the mean test–retest scores of participants who completed both administrations (*n* = 30), showed a strong positive relationship between the first and second administration of the NPED scale subdimensions and total score (Ease of Use: *r* = 0.89, *p* = 0.000; Impacts of EHRs on the Nursing: *r* = 0.83, *p* = 0.000; Concerns About EHRs: *r* = 0.72, *p* = 0.000; NPED Total: *r* = 0.90, *p* = 0.000).

### 4.3 Validity Analysis of the Scale

#### 4.3.1 CFA

CFA was conducted to test the conformity of the NPED scale with the theoretical structure proposed by Schenk et al. (2020). The covariance matrix was used to analyse item interactions, and the maximum likelihood estimation method was applied to examine the matrix. GFIs, including *χ* ^2^/df, RMSEA, RMR, SRMR, GFI, AGFI, CFI, NFI and NNFI, were evaluated to assess the fit of the data to the model. The CFA results are presented in Table 1.

**TABLE 1:** NPED scale descriptive statistics, reliability coefficients and item total score correlations (*n* = 211).

11‐Item NPED	Score mean ± SD	Item total score	*p*	Cronbach's Alpha coefficient	Internal consistency coefficient in case of item deletion
Item 1	3.70 ± 0.86	0.65			0.71
Item 2^a^	3.09 ± 0.96	0.27			0.77
Item 3	3.54 ± 0.88	0.62			0.72
Item 4	3.90 ± 0.84	0.68			0.71
Item 5	3.70 ± 0.97	0.63			0.72
Item 6^a^	3.59 ± 1.09	0.57	0.000^a^	0.75	0.73
Item 7^a^	3.74 ± 1.00	0.64			0.71
Item 8^a^	3.60 ± 0.98	0.53			0.73
Item 9^a^	3.93 ± 0.90	0.54			0.73
Item 10	2.79 ± 1.00	0.27			0.77
Item 11	3.54 ± 0.87	0.53			0.73
NPED total score	39.16 ± 5.59 (Min‐Max: 24.00–54.00)				
Ease of use	24.29 ± 3.90 (Min‐Max: 11.00–35.00)			0.71	
Impacts of EHRs on nursing	7.33 ± 1.89 (Min‐Max: 2.00–10.00)			0.76	
Concern about the EHR	7.54 ± 1.68 (Min‐Max: 2.00–10.00)			0.74	

^a^ Reverse items.

**FIGURE 1:**
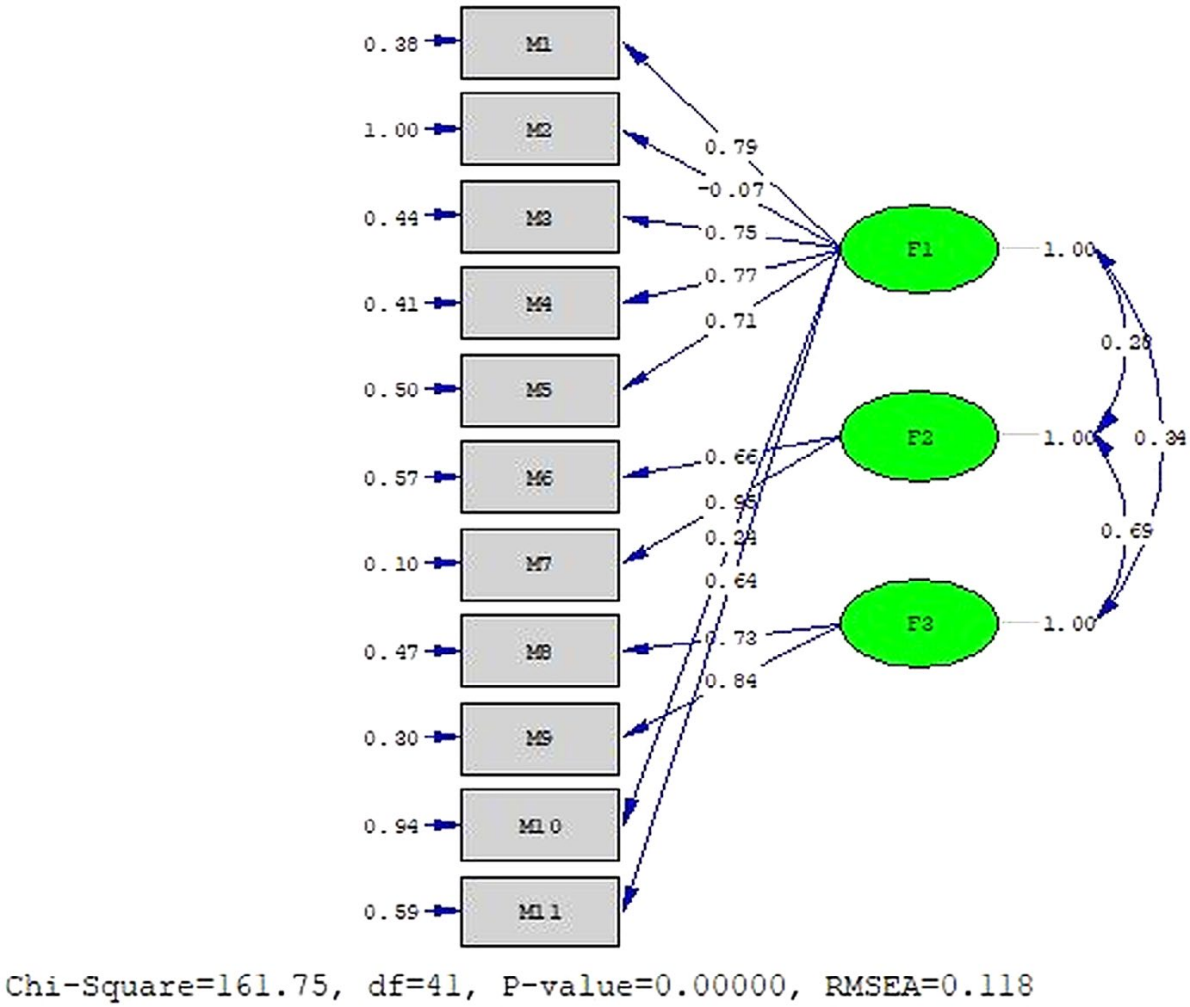
Nurses' perceptions of electronic documentation scale confirmatory factor analysis.

While *χ* ^2^/df and CFI indices indicated acceptable fit, RMSEA, RMR, SRMR, GFI, AGFI, NFI and NNFI indices fell below acceptable thresholds (Table 2). The three‐factor structure of the NPED scale was confirmed; however, the standardised estimates (maximum likelihood) for items 1, 3, 4, 5, 7, 8 and 9 were above the acceptable limit of 0.45, ranging between 0.53 and 0.90. Items 6 and 11 had standardised estimates of 0.43 and 0.41, respectively. Items 2 and 10 showed factor loadings of −0.07 and 0.24, respectively standardised estimates of 0.0047 and 0.056, respectively. Except for item 2, all t‐values for the NPED scale items were significant at *p* < 0.05. Overall, the factor loadings for the NPED scale model ranged between −0.07 and 0.95 (Figure 1).

**TABLE 2:** NPED scale confirmatory factor analysis fit index values and acceptable values.

Goodness of fit indices	NPED (three‐dimensional‐ original scale)	After removing one item and one modification (three‐dimensional‐ original scale)	After removing two items and one modification (three‐dimensional‐ original scale)	Acceptable values
*χ* ^2^/df	161.75/41 = 3.94	61.55/31 = 1.98	46.68/23 = 2.02	< 5
Root mean square error of approximation (RMSEA)	0.118	0.069	0.07	< 0.08
Root mean square residual (RMR)	0.096	0.064	0.056	< 0.08
Standardised root mean square residual (SRMR)	0.010	0.067	0.059	< 0.08
Goodness‐of‐fit index (GFI)	0.88	0.94	0.95	≥ 90 good fit
Adjusted‐goodness‐of‐fit index (AGFI)	0.80	0.90	0.91	≥ 90 good fit
Comparative fit index (CFI)	0.90	0.97	0.97	≥ 90 good fit
Normed fit index (NFI)	0.87	0.95	0.95	≥ 90 good fit
Nonnormed fit index (NNFI)	0.87	0.94	0.96	≥ 90 good fit

*Note:* *χ* ^2^/df, the ratio of chi‐squared to degrees of freedom.

**FIGURE 2:**
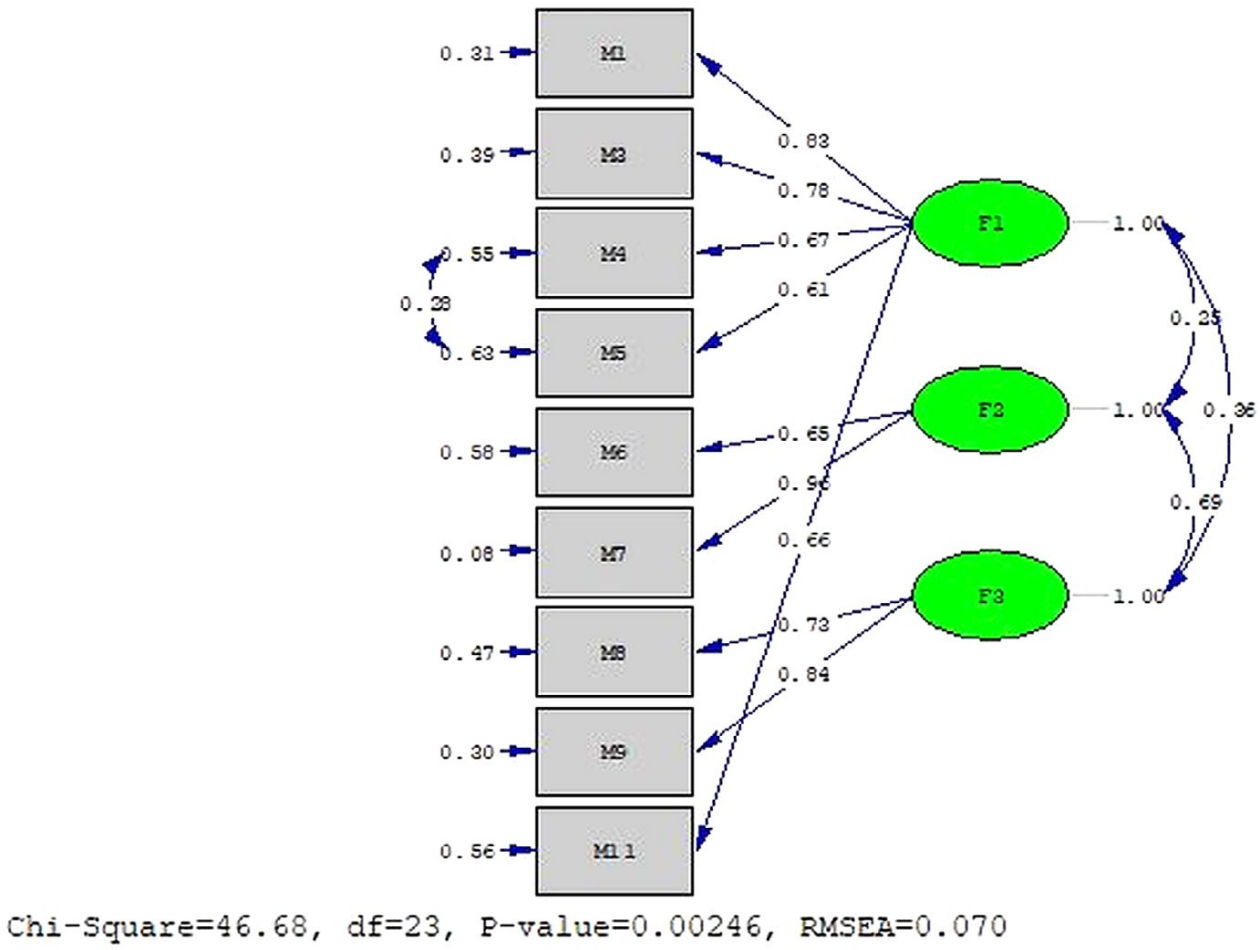
9‐item nurses' perceptions of electronic documentation scale confirmatory factor analysis.

As a result of the CFA, item 2 was removed due to its high error variance and nonsignificant *t* value. Following this removal, the CFA fit indices indicated that the RMR, SRMR, GFI, CFI, NFI and NNFI values were within acceptable ranges, whereas the *χ* ^2^/df, RMSEA and AGFI indices were below the acceptable thresholds. After performing a single modification to improve the GFIs, all indices reached acceptable values. Although the model's GFIs with one modification were satisfactory, the three‐factor structure of the 9‐item NPED scale was confirmed after removing item 10, which exhibited high error variance and a factor loading of 0.24. For the 9‐item NPED, a single modification was also performed to evaluate changes in the GFIs, and all indices were considered acceptable (Table 2). The standardised estimates (maximum likelihood) for items 1, 3, 4, 5, 7, 8 and 9 of the NPED scale exceeded the acceptable threshold of 0.45, ranging from 0.50 to 0.90. Conversely, the standardised estimates for items 6 and 11 were 0.43 and 0.41, respectively (ranging between 0.42 and 0.44 in the modified model). The *t* values for the 9‐item NPED scale were significant at the *p* < 0.05 level. Factor loadings for the NPED scale model ranged from 0.64 to 0.95 (0.61 to 0.96 in the modified model) (Figure 2).

#### 4.3.2 EFA

In EFA, the factor loadings, which represent the degree of association between a factor and an item, were used as a criterion to determine the allocation of items to specific factors. Scale items were assigned to the factor with which they exhibited the highest correlation. In this study, factors with an eigenvalue of at least 1 were included in the analysis.

The Kaiser–Meyer–Olkin (KMO) measure was employed to assess the suitability of the dataset for factor analysis, while Bartlett's Test of Sphericity was conducted to examine whether the variables were sufficiently intercorrelated. For the NPED, the KMO value was calculated as 0.788, and Bartlett's test yielded *χ* ^2^ = 848.341 (*p* < 0.000). After refining the scale to its final 9‐item version (NPED), the KMO value was recalculated as 0.781, with Bartlett's test yielding *χ* ^2^ = 766.705 (*p* < 0.000).

Upon examining the factor structure of the NPED, three subfactors were identified through factor analysis conducted with the scale items unconstrained. To assess the appropriateness of each item for factor analysis, the MSA (Measure of Sampling Adequacy) was calculated for each item. All items demonstrated MSA values > 0.50. However, items 2 and 10 aligned with different subdimensions compared to their placement on the original scale. In the EFA conducted following the removal of item 2 from the 11‐item NPED scale, item 10 was removed as it was distributed in a different subdimension from the original scale and was inconsistent with the items under the factor in which the item was included. In the EFA conducted after removing the 10th item from the 10‐item NPED, principal component analysis on the resulting 9‐item scale identified three factors with eigenvalues greater than 1. Following Varimax rotation, the first factor accounted for 39.48% of the variance, the second factor explained 22.72% and the third factor explained 11.27%, resulting in a total variance explained by the three factors of 73.48%. The internal consistency of the 9‐item NPED, as measured by Cronbach's alpha, was 0.795 (Table 3).

**TABLE 3:** 9‐item NPED scale exploratory factor analysis.

Items	Faktor 1	Faktor 2	Faktor 3
Item 1	0.834		
Item 3	0.823		
Item 4	0.789		
Item 11	0.766		
Item 5	0.728		
Item 9		0.847	
Item 8		0.846	
Item 6			0.882
Item 7			0.721
Eigenvalue	3.55	2.04	1.01
Explained variance	39.48	22.72	11.27
Cronbach's alpha coefficient	0.855	0.741	0.767
Total Cronbach's alpha coefficient	0.795		

#### 4.3.3 Known Groups Validity

For the known‐groups validity of the NPED, the criteria of proficiency in computer use and prior exposure to EHRs training were determined.

A significant difference (*p* < 0.05) was observed between nurses' proficiency in computer use and their total mean NPED scale scores. Nurses who rated themselves as ‘somewhat proficient’ in computer use had significantly lower mean scores compared to those who rated themselves as ‘proficient’ or ‘very proficient’ (Table 4).

**TABLE 4:** Comparison of nurses' perceptions of electronic documentation scale with some sociodemographic characteristics (*n* = 211).

Features	*n*	Mean ± SD	Test statistics
Proficiency in computer use			
Nonproficient (a)	8	35.87 ± 8.42	KW: −10.074
Somewhat proficient (b)	51	36.50 ± 5.10	*p*: 0.000*
Proficient (c)	131	39.98 ± 5.23	b<c = d**
Very proficient (d)	21	41.80 ± 5.16	
Previous knowledge of EHRs			
Yes	100	40.52 ± 5.33	*t*: 3.408
No	111	37.95 ± 5.56	*p*: 0.001*

Abbreviations: KW, Kruskal Wallis Test; *t*, independent samples *t*‐test.

*p < 0.05, **p value obtained as a result of Bonferroni correction *p* < 0.008.

Additionally, a significant difference (*p* < 0.05) was found between the status of prior EHRs training and the total mean NPED scale scores. Nurses who reported prior EHR training (‘yes’) had significantly higher mean scores than those who reported no prior training (‘no’). (Table 4).

## 5 Discussion

In this study, the psychometric properties of the Turkish version of the 11‐item NPED scale were assessed, including the basic distribution characteristics of the scale items, as well as its reliability and validity.

Following the language adaptation and piloting phases, the data were analysed for descriptive characteristics. In the 11‐item Turkish version of the NPED scale, it was determined that the mean scores for the total and all subscales were above the average. Furthermore, the total and subscales mean scores reported in the study conducted by Schenk et al. (2020) which included 153 nurses in a US hospital were found to be similar to the findings of the present study.

Regarding the reliability findings, the Cronbach's alpha reliability coefficient for the Turkish version of the 11‐item NPED scale was calculated as 0.75. The subdimensions, namely ease of use, impacts of EHRs on the nursing, and concerns about EHRs, had Cronbach's alpha coefficients of 0.71, 0.76 and 0.74, respectively. According to the literature, a Cronbach's alpha coefficient above 0.70 is considered ‘acceptable’ (Taber 2018). Therefore, the internal consistency of the entire scale and its subdimensions in this study is at an ‘acceptable’ level. Schenk et al. (2020) developed the NPED scale based on Masrom's (2007) adaptation of the Technology Acceptance Model (TAM). In Masrom's (2007) study involving 122 students, Cronbach's alpha coefficients for the scale subscales were reported as 0.89, 0.89 and 0.85, respectively. In the study conducted by Schenk et al. (2020) with the original (English) version of the NPED, the Cronbach's alpha coefficients for the subscales were reported as 0.84, 0.82 and 0.85, respectively. In their two‐factor NPED scale study in English, Brunelli et al. (2021) reported that the Cronbach's alpha reliability coefficient of the entire scale was 0.93, the Cronbach's alpha coefficient of the ease of use subdimension was 0.92, and the Cronbach's alpha coefficient of the concern about EHRs subdimension was 0.88. The reliability coefficients for the scale across both language versions (English and Turkish) demonstrate high internal consistency. The item‐total score correlation assesses the compatibility of each item within a scale with the overall scale (Hair et al. 2019). Item‐total and item‐subdimension correlations are expected to exceed 0.30 (Esin 2024). In this study, the item‐total score correlation for items 2 and 10 was 0.27. Internal consistency analysis revealed a slight increase in the Cronbach's alpha value (from 0.75 to 0.77) when these items were removed. Further analysis was conducted to determine whether these items aligned with the ease‐of‐use subscale. It was observed that removing item 2 increased the subscale's internal consistency coefficient from 0.71 to 0.81, and there was no correlation between item 2 and the subscale's total score. Conversely, when item 10 was removed, there was a minimal increase in the subscale's internal consistency coefficient, along with a moderate positive correlation between the item and the subscale's total score. Based on these findings, it was determined that item 2 did not measure the same construct as the ease of use subdimension.

The Hotelling's *T* ^2^ test was significant (*p <* 0.05), indicating that the mean scores of the scale items differed from each other. This finding suggests that participants did not respond uniformly to all items and that the items showed variability in responses.

The test–retest correlation, which assesses the stability of the NPED scale over time, was very high. The significant relationship between the two measurements (*p* < 0.01) demonstrates that the instrument is stable over time (Esin 2024). This finding suggests that NPED remain consistent over time, supporting the accuracy of their responses.

In the construct validity analysis of the scale, a CFA was conducted to assess its alignment with the original theoretical structure established by Schenk et al. (2020). The analysis revealed that, aside from the *χ* ^2^/df and CFI indices, the other GFIs did not meet acceptable thresholds. Furthermore, the factor loadings for items 2 and 10 were below the recommended minimum of 0.32, with factor loadings of −0.07 and 0.24, respectively. According to the literature, while opinions on the acceptable threshold for factor loadings vary, there is a consensus that a minimum factor loading of 0.32 is necessary for an item to be considered adequately related to its factor (Sallis et al. 2021; Tabachnick and Fidell 2021; Çokluk et al. 2025). A low factor loading suggests that the item is weakly associated with the underlying construct. Additionally, CFA requires that the *t* values for the relationships between latent variables and their observed indicators be statistically significant (Çokluk et al. 2025). In the construct validity test of the 11‐item NPED, it was determined that the *t*‐value for item 2 was not significant, and its error variance was high. Consequently, it was decided to remove item 2 from the scale. In Türkiye, a single software system, ‘Probel Software’, is predominantly used for EHRs (https://www.probel.com.tr/). This may explain why the statement in NPED, ‘Item 2: EHRs require too many steps to access data’, did not align with expectations in the CFA results. Nurses had likely adapted to the system and the use of a standardised software platform influenced their perceptions of this item. In the subsequent CFA with the remaining 10 items, the model—with one modification—demonstrated acceptable goodness‐of‐fit values. However, the error variance of item 10 was still high, with a factor loading of 0.24, below the recommended threshold of 0.32. Given this, item 10 (‘I am excited about using EHRs’) was also removed from the scale. It is believed that the high error variance and low factor loading for item 10 were due to cultural differences, as the statement may not evoke the same meaning in individuals with Turkish cultural context when translated into Turkish. After removing the 10th item with high error variance, the three‐factor structure of the 9‐item NPED scale was confirmed. Following a single modification to evaluate the changes in the GFIs, all indices achieved acceptable values. These findings demonstrate that the theoretical three‐factor structure of the 11‐item NPED, originally developed by Schenk et al. (2020), was confirmed (after removing two items).

In cross‐cultural scale adaptation studies, CFA is recommended as the first step. If CFA results fail to confirm the subdimension structure of the original scale or if the GFIs fall below acceptable thresholds, EFA is advised (Çokluk et al. 2025). In this study, EFA was conducted because the GFIs obtained from the CFA were suboptimal. The KMO values for the NPED scale were above 0.50, and the significance level of Bartlett's test results was *p* < 0.05, indicating that the data set was suitable for factor analysis. The EFA yielded three subfactors; however, items 2 and 10 were distributed into subdimensions that differed from those of the original scale. In the EFA, after removing item 2, item 10 aligned with a different subdimension from the original structure and was incompatible with the items within the associated factor. Consequently, item 10 was also removed. As a result of the EFA, it is considered sufficient for the total variance explained by the factors to fall within 40%–60% (Çokluk et al. 2025). In this context, the results of the EFA conducted after removing item 10 were above acceptable thresholds.

For the known‐groups validity of the original 11‐item NPED, nurses' proficiency in computer use and prior knowledge of EHRs were used as criteria. Nurses who rated their computer use as ‘proficient’ or ‘very proficient’ had significantly higher mean NPED scale scores compared to those who rated their proficiency as ‘somewhat proficient’. Similarly, nurses with prior training or information about EHRs also demonstrated higher mean scores on the NPED. These findings suggest that the scale effectively differentiates based on the expected criteria. This result aligns with expectations, as individuals with greater familiarity with computer use and the EHRs are likely to have more positive perceptions reflected in their NPED scale scores. Supporting this, Brunelli et al. (2021) reported that nurses with higher levels of computer proficiency exhibited significant differences in mean NPED scale scores. Additionally, Alhur (2023) emphasised the importance of nurses continuously improving their computer skills to stay updated on technological advancements, including EHRs. Similarly, Sibiya et al. (2023) reported that providing computer training to healthcare professionals plays a crucial role in ensuring the efficient operation of EHRs and is a key factor in their successful adoption.

### 5.1 Limitations

The study's findings were based solely on nurses working in a university hospital, and thus, they were evaluated within the context of the institution's specific working conditions and should not be generalised. The results are further limited to the participants' responses. In cross‐cultural scale adaptation studies, the variation in item meanings across cultures and the exclusive use of a single EHR software in Türkiye posed additional constraints. Another limitation is that EFA and CFA were performed using the same dataset. This approach may capitalise on sample‐specific characteristics and overestimate model fit. Furthermore, the limited number of validity and reliability studies, as well as the scarcity of descriptive research conducted using the NPED scale employed in this study, present challenges when interpreting and discussing the findings.

## 6 Conclusion

The Turkish version of the NPED scale was found to possess an acceptable level of validity and reliability. This psychometrically sound, self‐report tool is easy to administer and can be effectively used to assess nurses' perceptions of EHR implementation. Importantly, this study is the first to establish the validity and reliability of the scale within a sample of Turkish nurses. It is recommended that future studies evaluate the psychometric properties of the scale in different cultural contexts to enhance its applicability.

### 6.1 Scoring of the Instrument

The Turkish version of the scale is structured as a five‐point Likert scale (1: strongly disagree; 2: disagree; 3: undecided; 4: agree; 5: strongly agree). The scale comprises nine items distributed across three subscales: ease of use (items 1, 2, 3, 4, 9), impacts of EHRs on nursing (items 5 and 6), and concerns about EHRs (items 7 and 8). Notably, four of the items (items 5, 6, 7 and 8) include reverse‐scored expressions. The total possible score ranges from 9 to 45, with higher scores indicating more positive perceptions of EHRs.

## Author Contributions

S.P.Ç. and N.B. participated in the design of the study and in data analysis and interpretation. S.P.Ç. and N.B. contributed to data collection and manuscript writing. S.P.Ç. and N.B. have read and approved the final version of the manuscript and agreed with the order of presentation of the authors.

## Funding

The authors have nothing to report.

## Ethics Statement

This study adheres to the ethical principles outlined in the Declaration of Helsinki. For the adaptation of the measurement tool into Turkish, permission was obtained from Schenk et al. (2020) via e‐mail. For the implementation of the study, ethics committee permission (decision date: 20 December 2023; decision no: 20.478.486/2142) was obtained from the Health Sciences Ethics Committee of the Faculty of Medicine at Manisa Celal Bayar University, and permission was obtained from the institution where the data were collected.

## Conflicts of Interest

The authors declare no conflicts of interest.

## Data Availability

The data that support the findings of this study are available from the corresponding author upon reasonable request.

## Appendix A See Tables A1 and A2

**TABLE A1:** Nurses' Perceptions of Electronic Documentation Scale (Original English Version).

	Strongly disagree	Disagree	Neither agree nor agree	Agree	Strongly agree
The EHR is easy to use.					
2 The EHR requires too many steps to find data. (R)					
3 I have the support I need to succeed with the EHR.					
4 The EHR makes it easy to document patient care.					
5 The EHR improves patient care.					
6 The EHR interferes with the ‘art’ of nursing. (R)					
7 If I had my way, nurses would never have to use the EHR. (R)					
8 I have anxiety about using the EHR. (R)					
9 I dread using the EHR. (R)					
10 I am excited to use the EHR.					
11 I am confident that I am using the EHR effectively.					

*Note:* (R): Reverse items. The items should be coded to the same direction.

**TABLE A2:** Hemşirelerin Elektronik Dokümantasyon Algıları Ölçeği (Türkçe Versiyon).

	Kesinlikle katılmıyorum	Katılmıyorum	Ne katılıyorum ne katılmıyorum	Katılıyorum	Kesinlikle katılıyorum
Elektronik sağlık kaydının kullanımı kolaydır.					
2 Elektronik sağlık kaydında başarılı olmak için ihtiyacım olan desteğe sahibim.					
3 Elektronik sağlık kaydı hasta bakımının dokümantasyonunu kolaylaştırır.					
4 Elektronik sağlık kaydı hasta bakımını geliştirir.					
5 Elektronik sağlık kaydı hemşirelik sanatını engeller. (T)					
6 Eğer bana kalsaydı, hemşireler hiçbir zaman elektronik sağlık kaydını kullanmak zorunda kalmazlardı. (T)					
7 Elektronik sağlık kaydını kullanma konusunda endişelerim var. (T)					
8 Elektronik sağlık kaydını kullanmaktan korkuyorum. (T)					
9 Elektronik sağlık kaydını etkili bir şekilde kullandığıma eminim.					

*Note:* (T): Ters maddeler. Maddeler aynı yönde kodlanmalıdır.
